# Robotic-Assisted and Laparoscopic Bariatric Surgeries Still Have Clinically Comparable Outcomes

**DOI:** 10.1007/s11695-024-07368-1

**Published:** 2024-07-18

**Authors:** Clay L. Cashman, Swapnil V. Shah, Alexander G. Hall, Ryan W. Walters, Kalyana C. Nandipati

**Affiliations:** 1 School of Medicine, Creighton University, 2500 California Plaza, Omaha, NE 68178 USA; 2 Department of Clinical Research and Public Health, School of Medicine, Creighton University, 7710 Mercy Road, Education Building, Suite 502, Omaha, NE 68124 USA; 3 Department of Surgery, School of Medicine, Creighton University, 7710 Mercy Road, Education Building, Suite 501, Omaha, NE 68124 USA

**Keywords:** Bariatric surgery, Robotic surgery, MBSAQIP, Weight loss surgery, Sleeve gastrectomy, Gastric bypass, Duodenal switch

## Abstract

**Purpose:**

Bariatric surgery is considered the main treatment option for patients with severe obesity. The objective of our study is to compare intra- and postoperative outcomes between the robotic and laparoscopic approaches within the sleeve gastrectomy (SG), duodenal switch (DS), and Roux-en-Y gastric bypass (RYGB).

**Materials and Methods:**

The data from the MBSAQIP were collected for patients who underwent SG, DS, and RYGB between 2015 and 2021. The postoperative and procedural outcomes including 30-day morbidity and mortality as well as operation length were analyzed using regression models.

**Results:**

Our analysis included 1,178,886 surgeries with SG comprising the majority (70%) followed by RYGB (28%) and DS (1%). Other than a higher adjusted risk of unplanned reoperation for robotic RYGB (relative risk (RR) 1.07) and a statistically significant higher rate of postoperative wound disruption in robotic SG for robotic surgery (RR 1.56), there were no statistically significant between-approach differences including infection, wound disruption, death, or reoperation for DS, RYGB, or SG. Our data showed no significant difference in anastomotic leak rate between laparoscopic and robotic approaches in either the DS (*p* = 0.521) or RYGB (*p* = 0.800) procedures. Across our study period, the median operation lengths decreased significantly per year for both the robotic SG and DS.

**Conclusions:**

Robotic and laparoscopic bariatric surgical procedures have statistically similar 30-day patient outcomes. Robotic bariatric procedures do have significantly longer median operative times than laparoscopic procedures. The decision to use a robotic approach or laparoscopic approach should be made based upon surgeon experience and possibly cost.

**Graphical Abstract:**

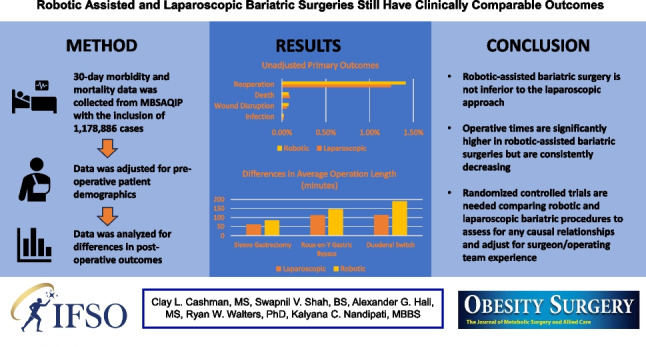

## Introduction

Obesity has become an epidemic in the United States with a prevalence rising to 41.9% in 2017 [1]. Bariatric surgery has been considered an important treatment option for patients with severe obesity (body mass index (BMI) > 35 with at least one related comorbidity or BMI > 40). The sleeve gastrectomy has been the most commonly performed bariatric surgery with the Roux-en-Y gastric bypass and duodenal switch also being regularly pursued surgical options [2]. The laparoscopic approach has been the standard of care and has a proven safety and efficacy record for all three procedures. However, due to challenges with the bariatric population such as limited intra-peritoneal space secondary to increased visceral fat content or hepatomegaly, surgeons continue to look towards alternative approaches, namely robotic surgery (RS).

Compared with the laparoscopic approach, the robotic platform offers several potential advantages including greater dexterity and precision with tissue manipulation [3]. Despite this theoretical advantage, controversy continues as to whether the robotic approach is similar in efficacy to the laparoscopic approach in practice. Numerous analyses exist comparing laparoscopic and robotic postoperative outcomes including studies which utilize large data registries such as the Metabolic and Bariatric Surgery Accreditation and Quality Improvement Program (MBSAQIP) database. Findings suggest little difference in mortality, potentially meaningful higher rates on key morbidity outcomes (such as surgical site infection rate and leak rate as calculated from composite variables) for RS, and significantly longer operation times for the robotic approach [4, 5]. However, questions about the maximum efficiency of the robotic platform persist, as does the possibility of further advances within the RS approach that may come from increased experience and more widespread usage. As the rate of usage of robotic bariatric surgery increases, continued assessment of the efficacies of each approach is critical to ensure patients are provided with the best possible outcomes.

The objective of our study is to compare intra- and postoperative outcomes between the robotic and laparoscopic approaches within the sleeve gastrectomy, duodenal switch, and Roux-en-Y gastric bypass.

## Materials and Methods

### Data Source and Case Selection

Data from the MBSAQIP between 2015 and 2021 were utilized to compare patients who underwent sleeve gastrectomy (SG), duodenal switch (DS), or Roux-en-Y gastric bypass (RYGB) and assess for differences in outcomes between the robotic-assisted and conventional laparoscopic-assisted approaches. The MBSAQIP data registry prospectively records more than 200 pre-, intra-, and postoperative variables using standardized definitions for all interventions performed at MBSAQIP-accredited centers in the United States and Canada; in 2020 and 2021, this represented 168,568 and 211,254 cases performed at 885 and 902 centers respectively [6]. MBSAQIP participant user data files include Health Insurance Portability and Accountability Act of 1996 (HIPPA) compliant patient-level data, and this retrospective cohort study was acknowledged as not human subjects research by the Institutional Review Board at Creighton University (record #2,003,469).

Cases meeting inclusion criteria were identified using Current Procedural Terminology (CPT) codes for SG (43,775), DS (43,845), and RYGB (43,644, 43,846,43,847) for the initial procedure. We then identified cases where the medical specialty of the physician performing the principal operative procedure was “Metabolic and Bariatric Surgeon,” or “General Surgeon” (combined these account for approximately 99% of cases), and identified whether the procedure was robotic or laparoscopic. These data were then merged with the 2015 to 2021 reoperation data in order to evaluate primary cohort outcomes such as reoperation.

### Outcomes

Primary postoperative outcomes were mortality, reoperation (overall and unplanned), and wound disruption. The primary procedural outcome was operation length (minutes). Year-over-year trends were also evaluated for unplanned reoperation and operative length given a sufficient sample size. Secondary outcomes included postoperative superficial incisional surgical site infections (SSI), deep incisional SSI, organ space SSI, pneumonia, unplanned intubation, pulmonary embolism, ventilator use, progressive renal insufficiency, acute renal failure, urinary tract infection, stroke/cerebrovascular accident (CVA), cardiac arrest requiring cardiopulmonary resuscitation (CPR), myocardial infarction, venous thrombosis requiring therapy, sepsis, septic shock, unplanned admission to the intensive care unit (ICU), readmission, and days to death from operation. We also evaluated postoperative anastomotic leak rate, but this variable was only available beginning in 2020. All outcomes measured were within 30 days of surgery.

### Covariate

For each surgery we abstracted age, biological sex, race, BMI (closest to bariatric surgery), smoking status, diabetes status, steroid use, chronic obstructive pulmonary disease (COPD), obstructive sleep apnea, previous foregut surgery, previous cardiac surgery, history of pulmonary embolism, hypertension, hyperlipidemia, history of deep vein thrombosis (DVT), American Society of Anesthesiologists (ASA) class, albumin levels, creatinine levels, renal insufficiency (pre-op), and dialysis.

### Analysis

Differences in primary outcomes, as well as pre- and postoperative characteristics between laparoscopic and robotic approaches in SG, DS, and RYGB were compared. Continuous variables are presented as the median and interquartile range (IQR) and compared statistically using the Mann–Whitney *U* test. Categorical variables are presented as frequency and percentage and compared statistically using chi-square tests. Primary outcomes were compared using separate log-binomial regression models for each procedure to quantify the unadjusted and adjusted risk for each 30-day outcome between RS and laparoscopic approaches. For the adjusted models, the included covariates varied; potential covariates were excluded if there were inadequate expected frequencies (< 5) or missing data. Risk ratios are reported alongside 95% confidence intervals, with ratios greater than 1 indicating a greater risk of outcome for the robotic-assisted group. Finally, Poisson and log-binomial regression models were estimated for each procedure to evaluate differences in operative length and unplanned reoperation, respectively, to evaluate year-over-year differences between approaches. All analyses were conducted using statistical analysis software (SAS) v. 9.4 with two-tailed *p* < 0.05 used to indicate statistical significance.

## Results

### Postoperative Outcomes

Our analysis included 1,178,886 surgeries, with SG comprising the majority (70%), followed by RYGB (29%) and DS (1%). Univariate analyses of case demographics and preoperative comorbid conditions are presented in Table 1. Analysis of our primary outcomes is presented in Tables 2 and 3. Overall, the observed unplanned reoperation rates were slightly higher in RS for all surgery types. However, this difference was only statistically significant within RYGB (unadjusted 2.4% RS vs 2.2% laparoscopic, *p* = 0.003). The adjusted rates of unplanned reoperation for this comparison demonstrated a 7% higher risk of unplanned reoperation (relative risk (RR) 1.07, 95% confidence interval (CI) 1.01–1.14,* p* = 0.035, Table 3). Similarly, the observed difference in wound disruption rates between RS and laparoscopic surgery was generally low (≈ 0.1%). Within the SG, RS unadjusted rates of wound disruption were 0.1% compared with laparoscopic rates of < 0.1%; however, this difference was statistically significant, and results of the adjusted model showed a 56% higher risk of wound disruption for RS (RR 1.56, 95% CI 1.18–2.06;* p* = 0.002, Table 3). There were no additional statistically significant between-approach differences in infection, wound disruption, death, or reoperation for DS, RYGB, or SG (Table 3). Univariate analyses for secondary postoperative outcomes are presented in Table 4. Postoperative anastomotic leak, which was only available for 2020 and 2021 (73,981 total robotic and 278,661 total laparoscopic cases), showed no between-approach difference for DS (18/1648 (1.1%) vs 31/3,428 (0.9%), *p* = 0.521) or RYGB (69/22,866 (0.3%) vs 265/82,745 (0.3%), *p* = 0.800) but a significantly higher rate for RS in SG (94/49,467 (0.2%) vs 266/192,488 (0.1%), *p* = 0.020).

**Table 1:** Demographics  Preoperative

	Duodenal switch			Roux-en-Y gastric bypass			Sleeve gastrectomy		
	Laparoscopic (*n* = 11,184)	Robot (*n* = 3864)	*p*	Laparoscopic (*n* = 291,495)	Robot (*n* = 44,386)	*p*	Laparoscopic (*n* = 726,343)	Robot (*n* = 102,614)	*p*
Female	8334 (74.5)	2924 (75.7)	0.236	239,722 (82.25)	37,161 (83.7)	< 0.001	581,767 (80.1)	82,968 (80.9)	< 0.001
Race			< 0.001			< 0.001			< 0.001
American Indian or Alaska Native	103 (0.9)	34 (0.9)	-	1637 (0.6)	220 (0.5)	-	2923 (0.4)	364 (0.4)	-
Asian	38 (0.3)	21 (0.5)	-	1535 (0.5)	216 (0.5)	-	3808 (0.5)	603 (0.6)	-
Black or African American	1322 (11.8)	833 (21.6)	-	43,995 (15.1)	7774 (17.5)	-	142,080 (19.6)	23,760 (23.2)	-
Native Hawaiian or other Pacific Islander	54 (0.5)	30 (0.8)	-	1105 (0.4)	80 (0.2)	-	1811 (0.3)	188 (0.2)	-
Race combinations with low frequency	3 (< 0.1)	3 (0.1)	-	67 (< 0.1)	23 (0.1)	-	164 (< 0.1)	44 (< 0.1)	-
Some other race	48 (0.43)	47 (1.2)	-	1097 (0.4)	367 (0.8)	-	3894 (0.5)	1035 (1.0)	-
Unknown/not reported	996 (8.9)	360 (9.3)	-	29,935 (10.3)	3442 (7.8)	-	68,497 (9.4)	8781 (8.6)	-
White	8619 (77.1)	2535 (65.6)	-	212,039 (72.7)	32,222 (72.6)	-	502,879 (69.2)	67,760 (66.0)	-
Current smoker within 1 year	938 (8.4)	322 (8.3)	0.917	21,112 (7.2)	2934 (6.6)	< 0.001	58,646 (8.1)	7676 (7.5)	< 0.001
Diabetes	3314 (29.6)	1108 (28.7)	0.260	90,034 (30.9)	13,503 (30.4)	0.048	158,550 (21.8)	22,666 (22.1)	.059
Obstructive sleep apnea	4598 (41.1)	1558 (40.3)	0.388	121,031 (41.5)	18,303 (41.2)	0.257	255,987 (35.2)	36,782 (35.9)	< 0.001
Pre-op GERD requiring medication	3613 (32.3)	1028 (26.6)	< 0.001	121,728 (41.8)	21,391 (48.2)	< 0.001	196,009 (27.0)	28,571 (27.8)	< 0.001
History of MI	146 (1.3)	54 (1.4)	0.667	3980 (1.4)	555 (1.3)	0.051	7925 (1.1)	1067(1.0)	0.138
History of COPD	229 (2.1)	59 (1.5)	0.042	4778 (1.6)	885 (2.0)	< 0.001	9859 (1.4)	1491 (1.5)	0.014
Hyperlipidemia	2587 (23.1)	874 (22.6)	0.514	78,409 (26.9)	12,293 (27.7)	< 0.001	154,755 (21.3)	22,426 (21.9)	< 0.001
Previous obesity surgery/foregut surgery	2399 (21.5)	896 (23.2)	0.024	39,535 (13.6)	7605 (17.1)	< 0.001	39,369 (5.4)	4697 (4.6)	< 0.001
Previous cardiac surgery	115 (1.0)	29 (0.8)	0.126	2830 (1.0)	423 (1.0)	0.721	7331 (1.0)	905 (0.9)	< 0.001
History of PE	207 (1.9)	71 (1.8)	0.958	4105 (1.4)	716 (1.6)	0.001	8531 (1.2)	1270 (1.2)	0.080
Pre-op vein thrombosis requiring therapy	241 (2.2)	108 (2.8)	0.023	5777 (2.0)	883 (2.0)	0.916	11,244 (1.6)	1594 (1.6)	0.896
ASA class			< 0.001			< 0.001			< 0.001
I	26 (0.2)	0 (0.0)	-	595 (0.2)	78 (0.2)	-	2420 (0.3)	344 (0.3)	
II	1334 (12.0)	538 (13.9)	-	49,629 (17.0)	7923 (17.9)	-	166,129 (23.0)	21,242 (20.7)	
III	9133 (82.0)	3118 (80.7)	-	228,856 (78.6)	34,882 (78.6)	-	531,805 (73.5)	77,077 (75.2)	
IV	641 (5.8)	207 (5.4)	-	12,023 (4.1)	1482 (3.3)	-	22,545 (3.1)	3800 (3.7)	
V	0 (0.0)	0 (0.0)	-	15 (< 0.1)	3 (< 0.1)	-	64 (< 0.1)	6 (< 0.1)	
Not assigned	4 (< 0.1)	1 (< 0.1)	-	72 (< 0.1)	7 (< 0.1)	-	491 (0.1)	45 (< 0.1)	
Pre-op therapeutic anticoagulation	366 (3.3)	145 (3.8)	0.156	8402 (2.9)	1492 (3.4)	< 0.001	19,386 (2.7)	2883 (2.8)	0.009
Pre-op renal insufficiency	64 (0.6)	19 (0.5)	0.560	1433 (0.5)	267 (0.6)	0.002	3233 (0.5)	473 (0.5)	0.476
	Median (IQR)								
Age	43.6 (35.9, 52.0)	42.8 (35.0, 51.0)	0.001	45.0 (36.8, 54.0)	46.0 (37.0, 54.2)	< 0.001	43.6 (35.0, 52.7)	43.1 (35.0, 52.1)	< 0.001
BMI	49.3 (43.5, 55.4)	50.0 (44.2, 56.7)	< 0.001	43.9 (39.7, 49.5)	43.5 (39.3, 49.2)	< 0.001	43.3 (39.5, 48.7)	43.6 (39.7, 49.0)	< 0.001
BMI_HIGH_BAR	51.2 (45.0, 57.9)	52.2 (46.1, 59.4)	< 0.001	45.9 (41.4, 51.8)	45.5 (41.0, 51.6)	< 0.001	45.1 (41.1 50.8)	45.3 (41.3, 51.1)	< 0.001
Albumin	4.0 (3.7, 4.3)	4.0 (3.8, 4.3)	0.960	4.1 (3.8, 4.3)	4.1 (3.8, 4.3)	0.119	4.1 (3.9, 4.4)	4.1 (3.8, 4.4)	< 0.001
Creatinine	0.8 (0.7, 0.9)	0.8 (0.7, 0.9)	0.152	0.8 (0.7, 0.9)	0.8 (0.7, 0.9)	0.529	0.8 (0.7, 0.9)	0.8 (0.7, 0.9)	0.453

Categorical variables are presented as frequency and percent, and between-group differences are tested with an omnibus chi-square test. Interval-level variables are reported as median values with quartiles 25 and 75, and between-group differences are tested using the Mann–Whitney *U* test

*GERD*, gastroesophageal reflux disease; *MI*, myocardial infarction; *COPD*, chronic obstructive pulmonary disease; *PE*, pulmonary embolism; *ASA*, American Society of Anesthesiologists; *BMI*, body mass index

**Table 2:** Primary Outcomes Unadjusted

	Duodenal switch			Roux-en-Y gastric bypass			Sleeve gastrectomy		
	Laparoscopic (*n* = 11,184)	Robot (*n* = 3864)	*p*	Laparoscopic (*n* = 291,495)	Robot (*n* = 44,386)	*p*	Laparoscopic (*n* = 726,343)	Robot (*n* = 102,614)	*p*
Infection									
Overall	8 (< 0.1)	1 (< 0.1)	0.463	87 (< 0.1)	11 (< 0.1)	0.656	76 (< 0.1)	11 (< 0.1)	0.871
Superficial	3 (< 0.1)	0 (< 0.1)	0.574	16 (< 0.1)	1 (< 0.1)	0.717	31 (< 0.1)	4 (< 0.1)	> 0.999
Deep incisional	3 (< 0.1)	0 (< 0.1)	0.574	11 (< 0.1)	2 (< 0.1)	0.686	7 (< 0.1)	0 (< 0.1)	> 0.999
Organ/space	2 (< 0.1)	1 (< 0.1)	0.426	61 (< 0.1)	8 (< 0.1)	0.859	38 (< 0.1)	7 (< 0.1)	0.496
Wound disruption	20 (0.2)	5 (0.1)	0.650	270 (0.1)	45 (0.1)	0.560	273 (< 0.1)	60 (0.1)	0.003
Death	37 (0.3)	6 (0.2)	0.082	400 (0.1)	51 (0.1)	0.265	454 (0.1)	56 (0.1)	0.382
Reoperation									
Overall	350 (3.1)	137 (3.6)	0.206	6718 (2.3)	1134 (2.6)	0.001	5738 (0.8)	859 (0.8)	0.115
Unplanned	336 (3.0)	132 (3.4)	0.216	6397 (2.2)	1075 (2.4)	0.003	5335 (0.7)	786 (0.8)	0.267

Categorical variables are presented as frequency and percent, *p-*value for between-group differences is for Fisher’s exact test

**Table 3:** Primary Outcomes: Univariate and Multiple Predictor Models

	Unadjusted		Adjusted	
	Risk ratio (95% CI)	*p*	Risk ratio (95% CI)	*p*
	Duodenal switch		Duodenal switch	
Unplanned reoperation	1.14 (0.93, 1.39)	0.203	1.11 (0.91, 1.37)	0.295
	Roux-en-Y gastric bypass		Roux-en-Y gastric bypass	
Unplanned reoperation	1.10 (1.04, 1.18)	0.003	1.07 (1.01, 1.14)	0.035
Wound disruption	1.09 (0.80, 1.50)	0.575	1.08 (0.79, 1.48)	0.650
Death	0.84 (0.63, 1.12)	0.232	0.83 (0.62, 1.11)	0.215
	Sleeve gastrectomy		Sleeve gastrectomy	
Unplanned reoperation	1.04 (0.97, 1.12)	0.270	1.05 (0.98, 1.13)	0.180
Wound disruption	1.56 (1.18, 2.06)	0.002	1.56 (1.18, 2.06)	0.002
Death	0.87 (0.66, 1.15)	0.338	0.87 (0.66, 1.15)	0.326

Ratios greater than 1 indicate higher outcome values for cases involving robotic surgery compared with laparoscopic. Adjusted models include covariates outlined in the “Materials and Methods” section; specific variables included as covariates in adjusted models vary by outcome and procedure depending on expected cell count

**Table 4:** Postoperative characteristics

	Duodenal switch			Roux-en-Y gastric bypass			Sleeve gastrectomy		
	Laparoscopic (*n* = 11,184)	Robot (*n* = 3864)	*p*	Laparoscopic (*n* = 291,495)	Robot (*n* = 44,386)	*p*	Laparoscopic (*n* = 726,343)	Robot (*n* = 102,614)	*p*
Post-op superficial incisional SSI	57 (0.5)	29 (0.8)	0.087	2615 (0.9)	183 (0.4)	< 0.001	1742 (0.2)	299 (0.3)	0.002
Post-op deep incisional SSI	19 (0.2)	6 (0.2)	0.848	474(0.2)	37 (0.1)	< 0.001	199 (< 0.1)	41 (< 0.1)	0.027
Post-op organ space SSI	128 (1.1)	64 (1.7)	0.015	1398 (0.5)	238 (0.5)	0.111	1267 (0.2)	305 (0.3)	< 0.001
Post-op pneumonia	53 (0.5)	13 (0.3)	0.265	1112 (0.4)	179 (0.4)	0.489	897 (0.1)	160 (0.2)	0.006
Unplanned intubation	38 (0.3)	19 (0.5)	0.185	614 (0.2)	90 (0.2)	0.114	622 (0.1)	115 (0.1)	0.008
Pulmonary embolism	24 (0.2)	20 (0.5)	0.003	509 (0.2)	70 (0.2)	0.424	642 (0.1)	126 (0.1)	0.001
Post-op ventilator	25 (0.2)	12 (0.3)	0.346	415 (0.1)	55 (0.1)	0.333	291 (< 0.1)	57 (0.1)	0.023
Progressive renal insufficiency	16 (0.1)	9 (0.2)	0.237	243 (0.1)	36 (0.1)	0.878	333 (0.1)	63 (0.1)	0.033
Acute renal failure	24 (0.2)	4 (0.1)	0.167	311 (0.1)	37 (0.1)	0.155	342 (0.1)	50 (0.1)	0.821
Post-op urinary tract infection	49 (0.4)	23 (0.6)	0.222	1447 (0.5)	196 (0.4)	0.123	2092 (0.3)	293 (0.3)	0.890
Stroke/CVA	5 (< 0.1)	1 (< 0.1)	0.613	38 (< 0.1)	6 (< 0.1)	0.934	86 (< 0.1)	21 (< 0.1)	0.023
Intra-op or post-op cardiac arrest requiring CPR	6 (0.1)	4 (0.1)	0.300	168 (0.1)	25 (0.1)	0.915	237 (< 0.1)	39 (< 0.1)	0.377
Intra-op or post-op myocardial infarction	4 (< 0.1)	1 (< 0.1)	0.771	110 (< 0.1)	11 (< 0.1)	0.180	173 (< 0.1)	19 (< 0.1)	0.296
Transfusion intra-op/post-op (72 h of surgery start time)	74 (0.7)	30 (0.8)	0.458	3477 (1.2)	350 (0.8)	< 0.001	3574 (0.5)	643 (0.6)	< 0.001
Post-op vein thrombosis requiring therapy	25 (0.2)	18 (0.5)	0.015	527 (0.2)	69 (0.2)	0.237	1447 (0.2)	223 (0.2)	0.226
Post-op sepsis	49 (0.4)	26 (0.7)	0.074	623 (0.2)	82 (0.2)	0.214	534 (0.1)	124 (0.1)	< 0.001
Post-op septic shock	37 (0.3)	13 (0.3)	0.958	389 (0.1)	52 (0.1)	0.377	227 (< 0.1)	52 (0.1)	0.002
Unplanned admission to ICU within 30 days	207 (1.9)	73 (1.9)	0.879	3111 (1.1)	413 (0.9)	0.008	3421 (0.5)	503 (0.5)	0.402
CDIFF	12 (0.2)	6 (0.2)	0.470	412 (0.2)	54 (0.1)	0.112	462 (0.1)	69 (0.1)	0.874
At least 1 readmission within 30 days of op	632 (5.7)	285 (7.4)	< 0.001	16,708 (5.7)	2929 (6.6)	< 0.001	19,798 (2.7)	2996 (2.9)	< 0.001
At least 1 intervention within 30 days of op	230 (2.1)	109 (2.8)	.006	6284 (2.2)	956 (2.2)	0.979	5227 (0.7)	791 (0.8)	0.070
	Median (Q1, Q3)								
Operation length (minutes)	114 (82, 168)	189 (148, 248)	< 0.001	112 (82, 149)	146 (113, 192)	< 0.001	62 (46, 85)	85 (65, 113)	< 0.001
Days to death from operation	5.0 (4.0, 11.0)	12 (9.0, 17.0)	0.138	7.0 (4.0, 150)	6.0 (4.0, 12.0)	0.512	9.0 (3.0, 17.0)	8.5 (3.0, 16.5)	0.926

*SSI*, surgical site infection; *CVA*, cerebrovascular accident; *CPR*, cardiopulmonary resuscitation; *ICU*, intensive care unit; *CDIFF*, clostridioides difficile

**Fig. 1:**
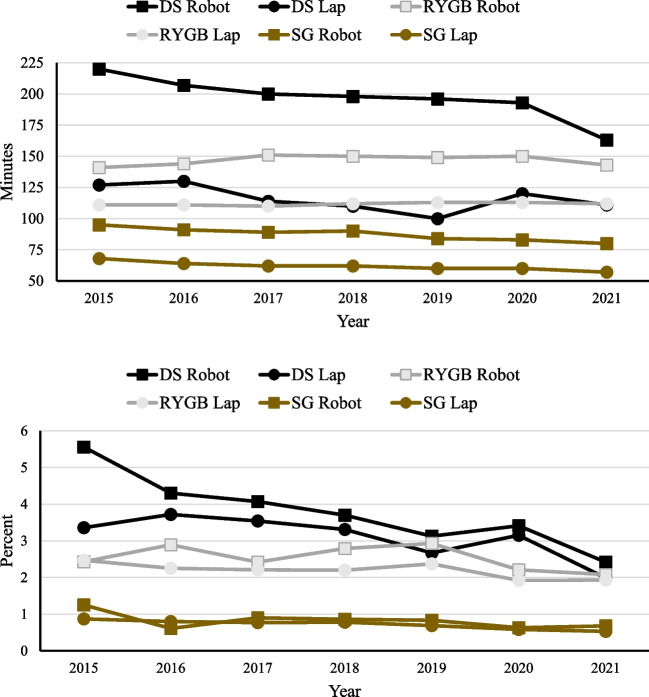
Median observed operation length in minutes (top) and unplanned reoperation rate in percent (bottom) between robotic and laparoscopic approaches by year for each procedure. Operation length and unplanned reoperation over time. *DS*, duodenal switch; *RYGB*, Roux-en-Y gastric bypass; *SG*, sleeve gastrectomy

Median observed operation length (minutes) and unplanned reoperation rate (percent) between robotic and laparoscopic approaches by year for each procedure are presented in Fig. 1. For the DS, there was an observed decreasing trend for the laparoscopic approach (observed median time decreased from 127 min in 2015 to 120 min in 2021, *p*_trend_ < 0.001); a similar pattern was observed for the robotic approach (observed median time decreased from 220 min in 2015 to 163 min in 2021, *p*_trend_ < 0.001) though it was decreasing to a greater extent (*p*_interaction_ < 0.001). The same pattern was observed for the SG, with median laparoscopic time (68 min in 2015 to 57 min in 2021, *p*_trend_ < 0.001) decreasing, and median RS time (95 min in 2015 to 80 min in 2021, *p*_trend_ < 0.001) decreasing to a greater extent (*p*_interaction_ < 0.001).

In modeling unplanned reoperation for the DS, there was an observed decreasing trend for laparoscopic surgery (3.36% in 2015 to 2.42% in 2021, *p*_trend_ < 0.003). A similar effect was observed for the robotic approach (5.56% in 2015 to 2.42% in 2021, *p*_trend_ < 0.007). This same pattern was observed for SG, with laparoscopic surgery (0.87% in 2015 to 0.53% in 2021, *p*_trend_ < 0.001) and RS (1.25% in 2015 to 0.68% in 2021, *p*_trend_ < 0.001) decreasing.

## Discussion

Bariatric surgery has become an effective treatment option for obesity and laparoscopic surgery has been the gold standard approach for several years. Recently, robotic surgery has emerged as a potential alternative to laparoscopic surgery. Overall, when adjusted for comorbid conditions and preoperative variables, the robotic approach is statistically similar to the laparoscopic approach in regards to major 30-day postoperative outcomes. This is consistently observed across all three procedures (sleeve gastrectomy, gastric bypass, and duodenal switch). However, statistically significant differences were noted with respect to the length of the operation and reoperations, especially in RYGB. Our results are unique in comparing all three major bariatric surgeries in a single large database study. Incorporating the most recent years of available MBSAQIP data many of our study findings, such as operative length and readmissions, showed similar rates to previously reported MBSAQIP studies. However, unplanned return to the operating room has been a point of controversy [3–5, 7–10].

Our results showed a statistically significant higher rate of unplanned reoperations within 30 days in robotic RYGB as well as a higher rate of postoperative wound disruption in robotic SG. These postoperative outcomes are consistent with prior literature [11]. Robotic surgery has shown similar trends in foregut surgery especially for hiatal hernia repairs in recent readmission database studies [12, 13]. Our data suggest that preoperative comorbid conditions such as COPD, prior foregut surgery, preoperative anticoagulation, and preoperative renal insufficiency, as well as characteristics including age, might be contributing slightly to higher reoperation rates in RYGB and higher readmission rates across all the robotic surgeries.

Postoperative bleeding and anastomotic-related complications, especially leaks, are considered the most common reasons for unplanned reoperations and morbidity. Our current dataset, which has leaks available from 2020, showed no significant difference between the laparoscopic and robotic approaches in either the DS or RYGB procedures. This is in part related to the lower total number of robotic DS and RYGB cases over 2 years (*n* = 1648 for DS and *n* = 22,866 for RYGB). Our results contrast with the retrospective study by Buchs N.C. et al. which showed that their robotic-assisted surgery group had a lower incidence of anastomotic leak (1.5% vs. 3.2%) and a shorter hospital stay (1.6 vs. 2.2 days) compared to the laparoscopic group [12, 13]. Our results showed similar leak rates between the procedures across platforms. Interestingly, SG (2020–2021 total *n* = 241,955) has a statistically significant higher rate (0.2% vs 0.1%) of leaks observed with the robotic approach. It is worth noting that, to our knowledge, this study includes the largest sample comparing leaks across the most common bariatric surgeries between approaches and is one of the initial reports comparing the leak rate for laparoscopic and robotic approaches across all major bariatric procedures in a large population database. As experience with RS has increased, the complications, especially leaks, have continued to show similar rates and trends when compared to the laparoscopic group.

The robotic approach has been proposed to be advantageous in patients with a high BMI classification as well as in technically difficult procedures like pancreatic surgery. In bariatric surgery, the DS is considered to be a technically challenging surgery, and robotic technology was adopted by some centers to show moderate benefits [14]. Our data showed that patients undergoing the robotic-assisted DS demonstrated a significantly higher rate of preoperative venous thrombosis requiring therapy, higher rates of prior foregut surgeries, and significantly higher BMIs. The higher BMIs in the DS group compared to the SG and RYGB could be explained by preference among bariatric surgeons whereas the choice to use the robotic-assisted approach in patients with higher BMIs could be explained by the better maneuverability offered by the robotic approach in cases with decreased intra-peritoneal space [11]. Our data suggest that utilizing a robotic approach for the DS is at least not statistically different from the laparoscopic approach with similar rates of leaks and unplanned reoperations. This trend is seen despite a patient population with a slightly higher severity of obesity, an increased need for anticoagulant therapy, and an increased rate of prior foregut procedures when compared to the laparoscopic patient population.

Our study does coincide with the current literature suggesting that robotic bariatric procedures have a longer average operation length [4, 7–9, 14]. In our data, RS had significantly longer operative times for all three procedures. The largest median difference was seen in the robotic duodenal switch which on average took 75 more minutes than the laparoscopic approach. This difference was smaller within the RYGB (robotic approach took 34 min longer on average) and sleeve gastrectomy (robotic approach took 23 min longer on average). Robotic-assisted RYGB did not show any decrease in median operative lengths across our data period. However, the median operative lengths for robotic-assisted SG and DS continued to decrease significantly per year during our data collection. This finding could be indicative of a learning curve associated with the robotic-assisted bariatric surgical techniques. Over time, the robotic approach should continue to improve and bring operative lengths closer to those with the laparoscopic approach, which should also bring down the cost associated with robotic surgery. In a recent publication by Ayesha P. Ng et al. utilizing the National Inpatient Sample (NIS) database, the robotic approach is associated with higher overall cost compared to the laparoscopic approach in select abdominal procedures (elective gastrectomy, cholecystectomy, colectomy, ventral hernia repairs, hysterectomy, and abdominoperineal resection) [15]. Interestingly, this cost discrepancy widened throughout their data period, though they remark that the average age and comorbidity burden increased across the period in the robotic group as well which is consistent with our study results. The increasing cost is an interesting finding that seems to contrast with our findings of decreasing operative lengths per year. A possible explanation is that robotic technology has seen continuous improvement since its implementation and it could be that the newer platforms, while allowing for more efficient operations length-wise, have a higher initial investment that has led to increasing cost of use. The per-year decrease in median operative times for the robotic-assisted SG and DS suggests that the argument of cost should be made longitudinally rather than be compared to one snapshot analysis of procedure-related costs which has been the norm currently.

It is also important to highlight the technical differences between the robotic and laparoscopic anastomotic procedures. The anastomotic technique in RS more commonly involves suturing compared to a stapled anastomotic technique in the laparoscopic approach. Our data does not include details about intraoperative technique, but it has been debated as one of the important factors in long-term outcomes including weight loss, stricture, and anastomosis-related complications. Literature reports have shown significantly lower stricture rates with robotic surgery [10].

Our findings of higher unplanned reoperations in the robotic RYGB and a higher rate of wound disruption in the robotic SG beg the question of the clinical reasons behind these findings. Anastomotic leaks and bleeding are considered the most important reasons for reoperation; however, in our data, neither of these was significantly different between approaches, making it harder to elucidate the reasons behind these findings. Some patient factors such as COPD were higher in the robotic SG group, and this may have contributed to a higher rate of wound disruption, an association suggested in prior literature [16]. However, the absolute differences between our significant findings are small, and the most important clinical takeaway from this data is that there is no clear inferiority between the robotic and laparoscopic approaches in bariatric surgery with regard to patient safety.

Our retrospective analysis does have limitations, one being the lack of long-term follow-up data (limited to 30 days). Another limitation is the lack of data concerning the surgeon and the operating room team. Much of the current discourse regarding the efficacy of laparoscopic vs. robotic surgery concerns the lag between technological advancement and gaining procedural experience. As there is no way to discern how experienced a surgeon or institution is in the MBSAQIP data, we are unable to comment on how surgical experience may have influenced our results. To elucidate if these extended operative times when compared to the laparoscopic approach are inherent to the robotic platform or a short-term finding that will resolve as procedural experience with this new technology improves, a comparative study should be conducted evaluating postoperative bariatric surgical outcomes and operative times in the robotic-assisted approach adjusting for surgeon experience. This limitation also impacts the external validity of our study, as it is a fair assumption to say that a surgeon and operating team that primarily performs one type of bariatric procedure will be more efficient and efficacious than a surgeon who performs a wider variety of procedures with less individual frequency. Our analysis and discussion are comparing all three bariatric procedures to assess for any apparent inferiority; however, if a surgeon is significantly more comfortable and experienced with one procedure and platform over others, then it is unlikely that our generalized results will apply to their specific circumstance. There also was not enough volume of data for the DS to allow for an adjusted analysis of postoperative wound disruption or mortality. As the MBSAQIP database continues to add years of data, the outcomes of DS can be better assessed.

One more limitation that is found in our study as well as a large portion of bariatric surgery literature is the retrospective design. The highest quality study for the assessment of causal relationships would be a randomized controlled trial utilizing a large patient population. This is of course difficult in bariatric surgery as ultimately it is patient preference that dictates the type of procedure performed (SG, DS, RYGB) after a discussion with their surgeon. However, randomization for the usage of the robotic or laparoscopic platform, independent of what bariatric procedure is decided upon, is much more feasible. With a large sample size and a longer follow-up period, this would provide invaluable data in the discussion of laparoscopic vs. robotic surgery outcomes. This could also remove the limitation arising from the lack of knowledge of surgeon expertise; as this would be a prospective study with known participants, these metrics could be easily accounted for. It may even reveal how significant differences in outcomes are between surgeons of different experiences.

## Conclusion

Robotic and laparoscopic bariatric surgical procedures have statistically similar 30-day patient outcomes. The robotic platform appears to be safe in the RYGB and DS despite a patient population with slightly increased rates of comorbidities and higher severities of obesity in the DS population. Robotic bariatric procedures do have significantly longer median operative times than laparoscopic procedures. Operative lengths have shown steady improvement with robotic surgery but remain high relative to the laparoscopic approach when utilizing the MBSAQIP database for comparison. Given the similarity in postoperative outcomes and continued improvement in operative times, the choice of approach should be dependent upon other factors such as surgeon experience and possibly overall cost.

## Data Availability

MBSAQIP participant use data files are available only to employees (surgeons, researchers, bariatric program staff, etc.) of MBSAQIP-participant centers.
